# Aldehyde Dehydrogenase 2 Activity Assessed Using the Acetaldehyde Breath Test Is Associated with Benign Pancreatic Disease and Pancreatic Cancer: A Case–Control Study

**DOI:** 10.3390/jcm15197543

**Published:** 2026-09-28

**Authors:** Yusuke Kamikihara, Shiroh Tanoue, Kohei Oda, Fumisato Sasaki, Yu Yamazato, Ryusuke Shibata, Issei Kojima, Yuko Morinaga, Makoto Hinokuchi, Shiho Arima, Tetsuya Idichi, Yuko Mataki, Takao Ohtsuka, Hiroshi Fujita, Chihaya Koriyama, Shinichi Hashimoto

**Affiliations:** 1 Digestive and Lifestyle Diseases, Graduate School of Medical and Dental Sciences, Kagoshima University, 8-35-1 Sakuragaoka, Kagoshima 890-8544, Japan; 2 Department of Epidemiology and Preventive Medicine, Graduate School of Medical and Dental Sciences, Kagoshima University, 8-35-1 Sakuragaoka, Kagoshima 890-8544, Japan; fiy@m.kufm.kagoshima-u.ac.jp; 3 Department of Digestive Surgery, Graduate School of Medical and Dental Sciences, Kagoshima University, 8-35-1 Sakuragaoka, Kagoshima 890-8544, Japan; idichitetuya@gmail.com (T.I.);; 4 Department of Gastroenterology, Izumi General Medical Center, 520 Myojincho, Izumi, Kagoshima 899-0131, Japan

**Keywords:** ALDH2, acetaldehyde breath test, pancreatic cancer, benign pancreatic disease, alcohol consumption

## Abstract

**Background/Objectives:** Aldehyde dehydrogenase 2 (ALDH2) detoxifies acetaldehyde, and impaired ALDH2 activity has been implicated in carcinogenesis. The acetaldehyde breath test (ABT) enables noninvasive functional assessment of ALDH2 activity, but its association with pancreatic disease remains unclear. We examined associations between ABT-assessed ALDH2 activity and pancreatic disease outcomes. **Methods:** This case–control study included 287 health checkup participants, 131 patients with benign pancreatic disease, and 65 patients with pancreatic cancer. Participants underwent ABT, and the acetaldehyde-to-ethanol (A/E) ratio was calculated. The ALDH2 inactive phenotype was defined as an A/E ratio ≥ 23.3. Multinomial logistic regression used health checkup participants as the reference. **Results:** The median A/E ratio was higher in patients with benign pancreatic disease and pancreatic cancer than in health checkup participants (21.7 and 24.2 vs. 13.6; *p* < 0.001). The ALDH2 inactive phenotype was associated with benign pancreatic disease (relative risk ratio [RRR], 3.55; 95% confidence interval [CI], 1.85–6.83) and pancreatic cancer (RRR, 4.82; 95% CI, 2.13–10.88). The highest A/E ratio category was also associated with benign pancreatic disease (RRR, 8.77; 95% CI, 3.59–21.39) and pancreatic cancer (RRR, 12.79; 95% CI, 4.41–37.09). Exploratory spline analyses showed associations between higher A/E ratios and pancreatic disease outcomes without clear evidence of nonlinearity. **Conclusions:** A higher ABT-derived A/E ratio was associated with benign pancreatic disease and pancreatic cancer in this selected case–control study. Prospective studies are needed to determine whether functional assessment of aldehyde metabolism provides clinically useful information for pancreatic disease risk assessment. **Trial registration number:** UMIN Clinical Trials Registry (UMIN-CTR), UMIN000052609; registered 1 November 2023.

## 1. Introduction

Pancreatic cancer remains one of the most lethal malignancies worldwide, with poor long-term survival despite advances in diagnosis and treatment. Established risk factors include smoking, obesity, diabetes mellitus, chronic pancreatitis, and pancreatic cystic lesions [1,2,3]. Alcohol consumption has also been associated with pancreatic cancer risk, particularly at moderate to heavy intake levels [4,5]. Because pancreatic cancer is often diagnosed at an advanced stage, identifying clinically feasible measures associated with pancreatic disease is an important challenge in general clinical and preventive care.

Benign pancreatic diseases are heterogeneous but clinically relevant in the context of pancreatic cancer risk. Chronic pancreatitis and pancreatic cystic lesions, including intraductal papillary mucinous neoplasms, are recognized as pancreatic cancer risk conditions [6,7,8,9,10,11,12]. However, the metabolic factors associated with benign pancreatic diseases remain incompletely understood. Previous studies have suggested associations between aldehyde dehydrogenase 2 (ALDH2) polymorphisms and alcoholic chronic pancreatitis [13,14], but evidence regarding functional ALDH2 activity and broader benign pancreatic disease remains limited. In particular, the relationship between functional aldehyde metabolism and pancreatic cystic lesions, including intraductal papillary mucinous neoplasms, has not been well defined. These lesions are increasingly detected in routine imaging and health checkup settings, and some require long-term surveillance because of their potential association with pancreatic cancer. Clarifying metabolic factors associated with benign pancreatic disease may therefore provide useful information for future risk assessment studies.

ALDH2 plays a central role in detoxifying acetaldehyde, a carcinogenic metabolite of ethanol [15]. The East Asian–specific ALDH2 rs671 variant markedly reduces enzymatic activity, leading to acetaldehyde accumulation [16]. This variant is strongly associated with alcohol-related upper aerodigestive tract cancers [17,18,19,20,21] and may influence cancer risk both directly through impaired acetaldehyde detoxification and indirectly through reduced alcohol consumption [22]. These complex effects support the importance of assessing functional ALDH2 activity in addition to genetic susceptibility.

The acetaldehyde breath test (ABT) is a noninvasive method for estimating ALDH2 activity in vivo by measuring the breath acetaldehyde-to-ethanol (A/E) ratio after ingestion of a small ethanol dose [23]. Previous studies have shown its utility in alcohol-related upper aerodigestive tract cancers [24,25]. However, whether ABT-derived functional ALDH2 activity is associated with benign pancreatic disease or pancreatic cancer has not been well characterized. Therefore, we evaluated the association between functional ALDH2 activity assessed using the ABT and pancreatic disease outcomes, including benign pancreatic disease and pancreatic cancer, using health checkup participants as the reference group.

## 2. Materials and Methods

### 2.1. Study Population

We conducted a case–control study including three groups: health checkup participants, patients with benign pancreatic disease, and patients with pancreatic cancer. Between November 2023 and August 2025, patients with suspected pancreatic cancer and patients with benign pancreatic disease were recruited from Kagoshima University Hospital. Recruitment of the pancreatic disease groups began after the initial approval of the study protocol on 30 August 2023. The use of the previously collected 2017 health checkup data in the present analysis was approved through a protocol amendment on 1 July 2025. In patients with pancreatic cancer, the ABT was performed during the diagnostic evaluation, when pancreatic cancer was strongly suspected based on imaging, but before histological confirmation and before the initiation of surgery or chemotherapy. Only patients whose diagnosis was subsequently confirmed histologically were included in the pancreatic cancer group. Histological tissue acquisition was performed by endoscopic ultrasound-guided fine-needle biopsy (EUS-FNB) using a 22-gauge Franseen needle, with three needle passes and 20 mL syringe suction; rapid on-site evaluation (ROSE) was not performed [26,27]. The health checkup group comprised individuals who underwent routine health checkups at Izumi General Medical Center, Kagoshima Prefecture, in 2017.

**Figure 1:**
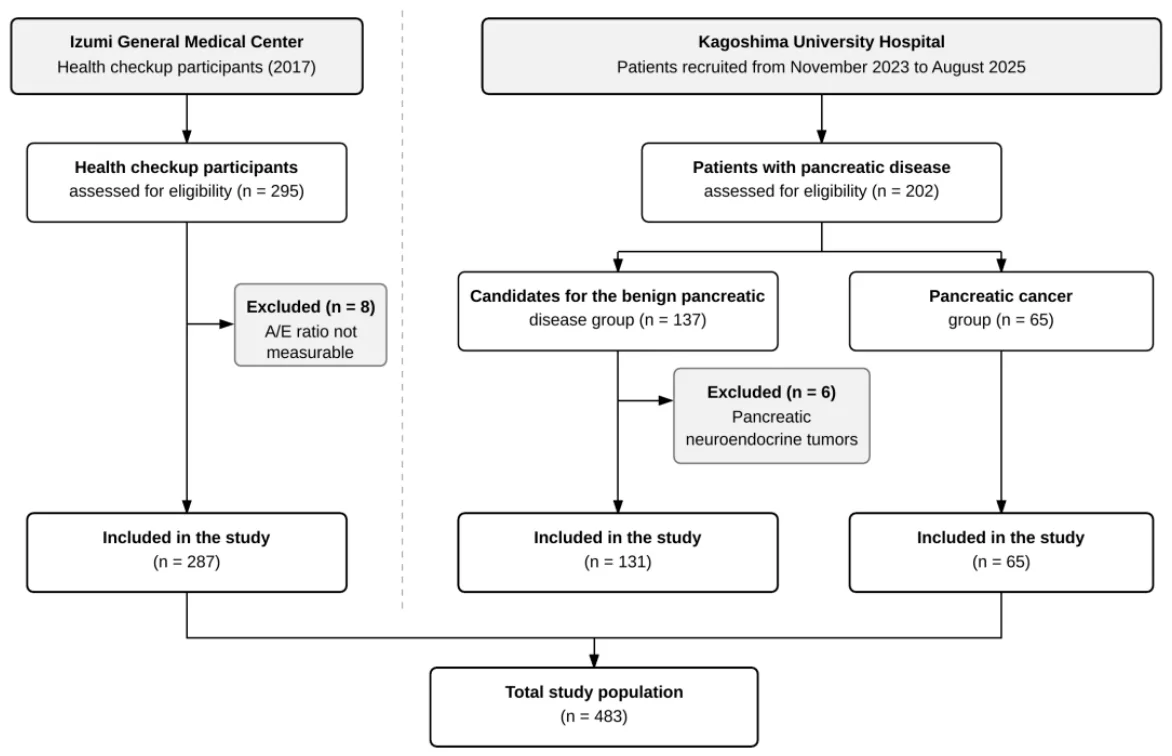
Flow diagram of participant selection and inclusion.

Benign pancreatic diseases included intraductal papillary mucinous neoplasm, chronic pancreatitis, pancreatic cyst, main pancreatic duct dilatation, autoimmune pancreatitis, serous cystic neoplasm, pancreatic divisum, mucinous cystic neoplasm, and other benign pancreatic conditions. The detailed distribution of diagnoses in the benign pancreatic disease group is shown in Supplementary Table S2. Clinical background and lifestyle information were obtained from medical records and standardized questionnaires, including age, sex, body mass index, daily alcohol consumption, smoking history, diabetes mellitus, history of malignancy, and family history of pancreatic cancer. A family history of pancreatic cancer was defined as a history in first-degree relatives, and a history of malignancy was defined as any prior malignancy other than pancreatic cancer. Health checkup participants were selected as a pragmatic reference group because both clinical groups consisted of patients with pancreatic disease, and a population not selected on the basis of known pancreatic disease was needed to contextualize the A/E ratios observed in the disease groups. These participants had undergone the ABT using the same protocol and measurement system as the patient groups. The participant selection process is shown in Figure 1.

### 2.2. Acetaldehyde Breath Test

Breath acetaldehyde and ethanol concentrations were measured using the ABT, as previously described [23]. Participants consumed 100 mL of 0.5% ethanol solution in a single ingestion, and end-tidal breath samples were collected 1 min after ingestion using dedicated gas bags. Breath acetaldehyde and ethanol concentrations were measured by high-sensitivity gas chromatography using the AERoChrome analyzer (Nissha FIS, Inc., Osaka, Japan) [28]. The gas bags were designed for end-tidal breath collection to reduce contamination from physiological dead space. Approximately 100 mL of end-tidal breath was collected during a single exhalation and used for measurement. The ethanol solution was prepared using an alcoholic beverage with low acetaldehyde content. The A/E ratio was automatically calculated. Based on a previous validation study of 111 Japanese healthy volunteers, an A/E ratio ≥ 23.3 was used to define the ABT-derived ALDH2 inactive phenotype; this cutoff identified carriers of the ALDH2*2 allele with 100% sensitivity, 92.5% specificity, and 96.4% overall accuracy [23]. The same ABT protocol, ethanol dose, sampling timing, and AERoChrome measurement system were used for health checkup participants and patient groups.

### 2.3. Exposure Assessment

Daily alcohol consumption was calculated as grams of ethanol per day using standard Japanese drink conversion factors and assessed for the preceding 10 years. One 108 mL serving of shochu was considered equivalent to 27 g of ethanol; one 180 mL serving of sake, 25 g; one large 720 mL bottle of beer, 25 g; one 80 mL glass of wine, 10 g; and one 28.5 mL shot of whiskey, 12.5 g. Alcohol consumption was analyzed as a continuous variable in the main multivariable models and was additionally categorized as 0 g/day, >0–<46 g/day, and ≥46 g/day. Smoking status was assessed using the Brinkman Index, calculated as the number of cigarettes smoked per day multiplied by years of smoking; a smoking history was defined as a Brinkman Index ≥ 600. Diabetes mellitus was defined based on medical records, self-reported history, or current treatment for diabetes.

### 2.4. Statistical Analyses

Continuous variables are presented as medians with interquartile ranges, and categorical variables as numbers with percentages. Baseline characteristics were compared using the Kruskal–Wallis test for continuous variables and the chi-squared or Fisher’s exact test for categorical variables, as appropriate.

Multinomial logistic regression analysis was performed to evaluate the association between ALDH2 inactive phenotype and pancreatic disease outcomes. The health checkup group was used as the reference category, and relative risk ratios (RRRs) with 95% CIs were estimated for benign pancreatic disease and pancreatic cancer. The primary model was adjusted for age, sex, alcohol consumption, smoking status, and diabetes mellitus. Sex-stratified analyses were exploratory, and sex was excluded from the adjustment variables. These covariates were prespecified based on their established or plausible associations with pancreatic disease and aldehyde metabolism.

To examine the association between a higher A/E ratio and pancreatic disease outcomes beyond the established cutoff for ALDH2 inactive phenotype, the A/E ratio was categorized into three groups: <23.3, 23.3–<36.9, and ≥36.9. The cutoff value of 36.9 corresponded to the median A/E ratio among participants with an A/E ratio ≥ 23.3. Multinomial logistic regression was performed using the lowest A/E ratio category as the reference.

To evaluate the A/E ratio as a continuous exposure without assuming linearity, restricted cubic spline analyses were performed as exploratory analyses. Separate logistic regression models were constructed for overall pancreatic disease, defined as benign pancreatic disease or pancreatic cancer, versus the health checkup group; benign pancreatic disease versus the health checkup group; and pancreatic cancer versus the health checkup group. Three knots were placed at the 10th, 50th, and 90th percentiles of the A/E ratio distribution, corresponding to 4.6, 17.4, and 50.6, respectively. ORs were estimated using an A/E ratio of 23.3, the established cutoff for defining the ALDH2 inactive phenotype, as the reference value. These models were adjusted for age, sex, alcohol consumption, smoking status, and diabetes mellitus. Overall associations of the spline terms and evidence of nonlinearity were assessed using Wald tests.

All statistical analyses were performed using Stata/SE version 18.0 (StataCorp, College Station, TX, USA). All *p*-values were two-sided, and *p* < 0.05 was considered statistically significant. Missing data were not imputed. Descriptive analyses were based on available data, and multivariable analyses were performed using complete cases for all variables included in each model.

To explore heterogeneity within the benign pancreatic disease group, additional post hoc multinomial logistic regression analyses were performed separately for patients with IPMN and those with non-IPMN benign pancreatic diseases. The health checkup group was used as the reference category, and the models were adjusted for the same covariates as the primary analysis.

To address the substantial age difference between the study groups, an additional post hoc sensitivity analysis was performed using health checkup participants aged ≥60 years as a more age-comparable reference group, while retaining all patients with benign pancreatic disease and pancreatic cancer. The age threshold was selected to improve age comparability while retaining an adequate number of reference participants. Multinomial logistic regression was performed using the same covariates as in the primary analysis, without introducing additional adjustment variables. The results of this analysis are presented in Supplementary Table S4.

No formal a priori sample size calculation or power analysis was performed. The sample size was determined by the number of eligible participants available during the study periods.

## 3. Results

### 3.1. Baseline Characteristics of the Study Population

A total of 483 participants were included: 287 health checkup participants, 131 patients with benign pancreatic disease, and 65 patients with pancreatic cancer. All 65 pancreatic cancer cases were histologically confirmed pancreatic ductal adenocarcinomas. Among the 65 patients with pancreatic cancer, the stage distribution according to the eighth edition of the Union for International Cancer Control (UICC) TNM classification was as follows: stage IA, 4 patients (6.2%); stage IB, 3 (4.6%); stage IIA, 24 (36.9%); stage IIB, 10 (15.4%); stage III, 5 (7.7%); and stage IV, 19 (29.2%). Regarding resectability, 34 patients (52.3%) had resectable disease, 4 (6.2%) had borderline resectable disease, and 27 (41.5%) had unresectable disease. Baseline characteristics are shown in Table 1. Participants in the disease groups were older than health checkup participants and differed in sex distribution, body mass index, alcohol consumption, smoking history, diabetes mellitus, history of malignancy, and family history of pancreatic cancer. The median A/E ratio was higher in the benign pancreatic disease and pancreatic cancer groups than in the health checkup group (21.7 and 24.2 vs. 13.6; *p* < 0.001). The prevalence of the ALDH2 inactive phenotype, defined as an A/E ratio ≥ 23.3, was also higher in the benign pancreatic disease and pancreatic cancer groups than in the health checkup group (47.3% and 50.8% vs. 30.7%; *p* < 0.001). Sex-stratified baseline characteristics are shown in Supplementary Table S1. In both men and women, patients with benign pancreatic disease and pancreatic cancer were older and had higher A/E ratios than health checkup participants. The benign pancreatic disease group included 74 patients with intraductal papillary mucinous neoplasm (IPMN) and 57 patients with non-IPMN benign pancreatic diseases. The number of patients with each specific diagnosis is presented in Supplementary Table S2.

RRR, relative risk ratio; CI, confidence interval; A/E ratio, acetaldehyde-to-ethanol ratio; DM, diabetes mellitus; ALDH2, aldehyde dehydrogenase 2; BI, Brinkman Index. Multinomial logistic regression analysis was performed with the screening group as the reference category. The model was adjusted for age, sex, alcohol consumption (g/day), smoking status (BI > 600), and diabetes mellitus. A/E ratio ≥ 23.3 was used to define the ALDH2 inactive phenotype.

A/E ratio, acetaldehyde-to-ethanol ratio; BMI, body mass index; DM, diabetes mellitus; IQR, interquartile range; HC, health checkup. Alcohol consumption categories were defined as 0 g/day, >0 to <46 g/day, and ≥46 g/day based on daily ethanol intake. Smoking status was defined as a Brinkman Index ≥ 600. Family history of pancreatic cancer was defined as a history in first-degree relatives. History of malignancy was defined as a history of malignancy excluding pancreatic cancer.

Continuous variables are presented as median (interquartile range) and were compared using the Kruskal–Wallis test. Categorical variables are presented as number (percentage) and were compared using the chi-square test or Fisher’s exact test, as appropriate. Fisher’s exact test was used for family history of pancreatic cancer.

### 3.2. Association Between ALDH2 Inactive Phenotype and Pancreatic Disease Outcomes

Multinomial logistic regression analysis was performed using the health checkup group as the reference category (Table 2). After adjustment for age, sex, alcohol consumption, smoking status, and diabetes mellitus, the ALDH2 inactive phenotype was associated with both benign pancreatic disease and pancreatic cancer. The adjusted RRRs were 3.55 (95% CI, 1.85–6.83) for benign pancreatic disease and 4.82 (95% CI, 2.13–10.88) for pancreatic cancer. In sex-stratified analyses, the ALDH2 inactive phenotype was associated with benign pancreatic disease in men and women. Sex-stratified analyses were exploratory, and no formal interaction test was performed; therefore, these findings should not be interpreted as evidence of sex-specific effect modification.

In the additional analyses, the ALDH2 inactive phenotype was associated with IPMN (adjusted RRR, 4.09; 95% CI, 1.64–10.21) and non-IPMN benign pancreatic diseases (adjusted RRR, 3.45; 95% CI, 1.62–7.33), compared with the health checkup group (Supplementary Table S3).

In the post hoc sensitivity analysis using health checkup participants aged ≥60 years as a more age-comparable reference group, 241 participants with complete data were included: 50 health checkup participants, 126 patients with benign pancreatic disease, and 65 patients with pancreatic cancer. Their median ages were 66 years (IQR, 61–69), 71 years (IQR, 66–76), and 73 years (IQR, 67–78), respectively. The ABT-defined ALDH2 inactive phenotype remained associated with benign pancreatic disease (adjusted RRR, 2.98; 95% CI, 1.32–6.74) and pancreatic cancer (adjusted RRR, 3.98; 95% CI, 1.60–9.92) (Supplementary Table S4).

### 3.3. Association Between the A/E Ratio and Pancreatic Disease Outcomes

To examine associations beyond the established cutoff for the ALDH2 inactive phenotype, the A/E ratio was categorized into three groups: <23.3, 23.3–<36.9, and ≥36.9 (Table 3). Compared with participants with an A/E ratio < 23.3, those in the highest A/E ratio category had markedly higher RRRs for benign pancreatic disease (RRR, 8.77; 95% CI, 3.59–21.39) and pancreatic cancer (RRR, 12.79; 95% CI, 4.41–37.09). The intermediate category showed modest but non-significant increases in RRRs.

**Figure 2:**
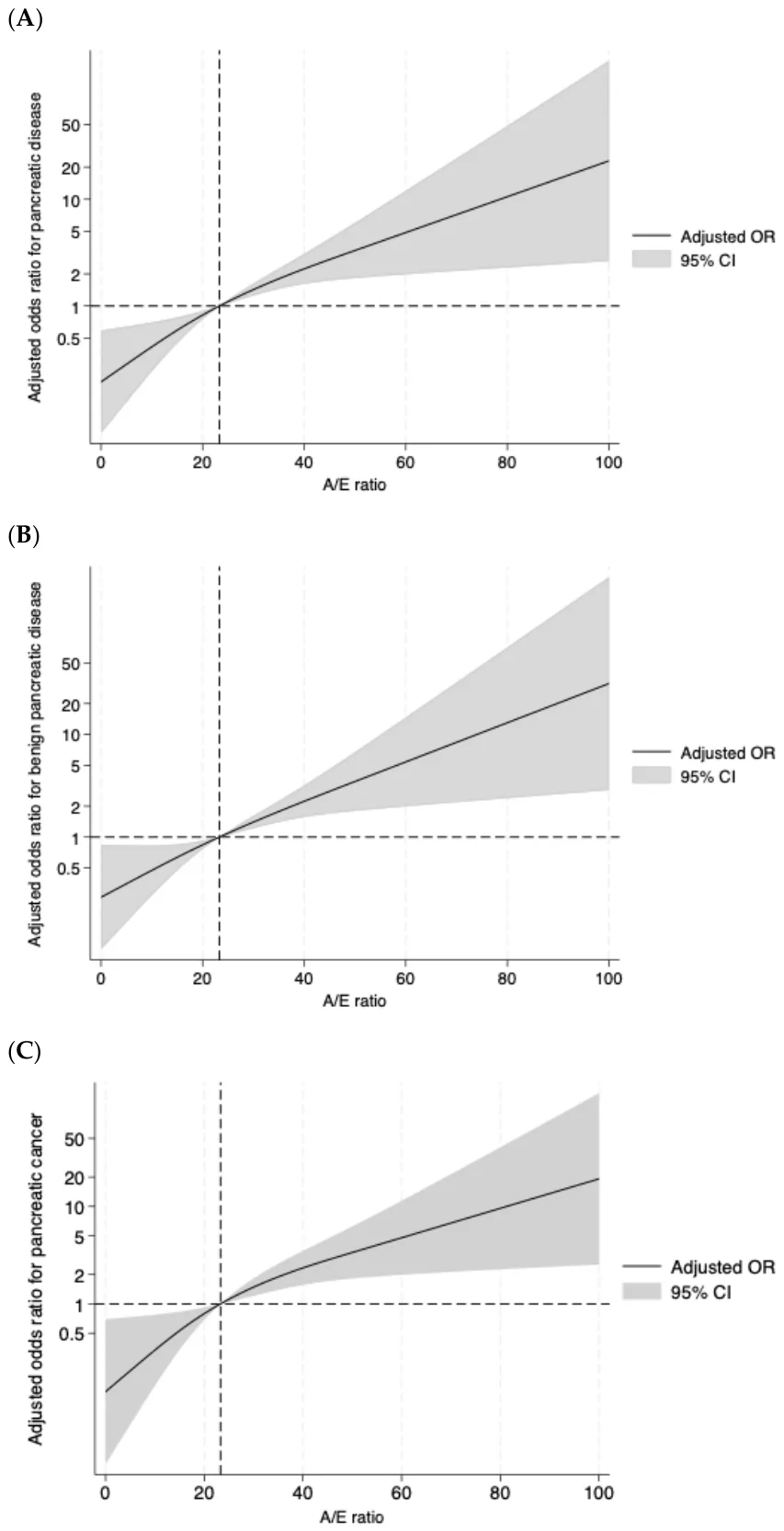
Restricted cubic spline analyses of the association between the A/E ratio and pancreatic disease outcomes: (**A**) Overall pancreatic disease, including benign pancreatic disease and pancreatic cancer, versus the health checkup group. (**B**) Benign pancreatic disease versus the health checkup group. (**C**) Pancreatic cancer versus the health checkup group.

Restricted cubic spline analyses were performed to evaluate the A/E ratio as a continuous exposure (Figure 2). In the analysis of overall pancreatic disease versus the health checkup group, the A/E ratio was significantly associated with overall pancreatic disease after adjustment for age, sex, alcohol consumption, smoking status, and diabetes mellitus (overall association, *p* < 0.001), without significant evidence of nonlinearity (*p* for nonlinearity = 0.3724). Similar findings were observed for benign pancreatic disease (overall association, *p* < 0.001; *p* for nonlinearity = 0.7149) and pancreatic cancer (overall association, *p* = 0.0002; *p* for nonlinearity = 0.2315). These findings suggest that a higher A/E ratio was associated with pancreatic disease outcomes when modeled as a continuous exposure, although no clear nonlinear pattern was observed.

Adjusted odds ratios were estimated using logistic regression models with an A/E ratio of 23.3 as the reference value. The models were adjusted for age, sex, alcohol consumption, smoking status, and diabetes mellitus. Solid lines indicate adjusted odds ratios, and shaded areas indicate 95% confidence intervals. Knots were placed at the 10th, 50th, and 90th percentiles of the A/E ratio distribution, corresponding to 4.6, 17.4, and 50.6, respectively. A/E ratio: acetaldehyde-to-ethanol ratio; OR: odds ratio; CI: confidence interval.

## 4. Discussion

In this case–control study of health checkup participants, patients with benign pancreatic disease, and patients with pancreatic cancer, impaired functional ALDH2 activity assessed using the ABT was associated with both benign pancreatic disease and pancreatic cancer after adjustment for major covariates. The A/E ratio was higher in both disease groups than in the health checkup group, and the ALDH2 inactive phenotype was associated with increased RRRs for benign pancreatic disease and pancreatic cancer. These findings suggest that impaired aldehyde metabolism may be related to pancreatic disease status, although causality cannot be inferred from this study design.

Compared with previous genotyping-based studies of ALDH2 and pancreatic cancer, the present study has the distinctive feature of assessing functional ALDH2 activity using the ABT-derived A/E ratio not only as a binary phenotype but also as a continuous measure. This approach allowed us to evaluate dose–response patterns across the A/E ratio distribution and to extend the assessment to benign pancreatic disease, although the smaller pancreatic cancer sample limits the strength of inference.

The inclusion of a benign pancreatic disease group is an important feature of this study. Benign pancreatic diseases are heterogeneous, but chronic pancreatitis and pancreatic cystic lesions, including intraductal papillary mucinous neoplasms, are clinically relevant pancreatic cancer risk conditions [6,7,8,9,10,11,12]. Prior studies have suggested associations between ALDH2 polymorphisms and alcoholic chronic pancreatitis [13,14], but evidence regarding functional ALDH2 activity and broader benign pancreatic disease has been limited. Our findings raise the possibility that impaired aldehyde metabolism may be associated with pancreatic disease before overt malignancy. However, the benign pancreatic disease group included clinically distinct conditions that differed in their pathophysiology, relationship with alcohol consumption, inflammatory activity, and general clinical or metabolic status. These differences may have influenced the A/E ratio. Therefore, the pooled association observed for benign pancreatic disease should not be interpreted as evidence of an association with each individual pancreatic disorder, and adequately powered disease-specific studies are needed.

We also explored the A/E ratio beyond a binary ALDH2 phenotype classification. Participants in the highest A/E ratio category had substantially higher RRRs for benign pancreatic disease and pancreatic cancer than those with an A/E ratio < 23.3; however, the intermediate category was not significantly associated with either outcome. Because the cutoff value of 36.9 was derived from the present study population, we additionally modeled the A/E ratio as a continuous exposure using restricted cubic splines. These analyses showed overall associations of the A/E ratio with pancreatic disease outcomes without clear evidence of nonlinearity. Because several related secondary analyses were conducted without formal adjustment for multiple comparisons, the findings require external validation in prospective cohorts. Thus, the spline findings support the exploratory evaluation of the A/E ratio as a continuous measure but do not establish a clinically actionable threshold or its predictive utility.

Alcohol consumption remains important when interpreting the relationship between ALDH2 activity and pancreatic disease. Prior epidemiological studies have suggested that pancreatic cancer risk is increased mainly among individuals with moderate to heavy alcohol consumption [4,5,29]. In addition, ALDH2 rs671 may influence pancreatic carcinogenesis through both direct effects related to impaired acetaldehyde detoxification and indirect effects mediated by reduced alcohol consumption [22]. In the present study, alcohol consumption during the preceding 10 years was retrospectively assessed and adjusted for as a continuous variable in the multivariable models. However, detailed information on lifetime alcohol exposure, drinking patterns, and changes in consumption following disease onset was not available. Moreover, because ALDH2 activity may influence drinking behavior and pancreatic disease may subsequently alter alcohol consumption, this adjustment could not fully distinguish confounding, mediation, and reverse causation. Phenotype-stratified alcohol analyses were not emphasized because of the limited number of pancreatic cancer cases and the resulting statistical instability. Therefore, the relationship among ALDH2 activity, alcohol exposure, and pancreatic disease should be interpreted cautiously and examined in larger prospective studies.

The ABT provides a noninvasive functional assessment of aldehyde metabolism. Previous studies have shown that it can identify ALDH2*2 allele carriers with high accuracy and may be useful in alcohol-related upper aerodigestive tract cancers [23,24,25], whereas alcohol flushing questionnaires and ethanol patch tests have practical limitations [30,31,32,33,34]. Because the ABT-derived A/E ratio reflects the metabolic response to a small ethanol load, it may provide information beyond self-reported drinking behavior or genotype alone, while also reflecting other physiological factors. However, the present findings do not establish the ABT as a screening or risk stratification tool for pancreatic disease, and prospective validation is required.

This study has several limitations. First, the case–control design precludes causal inference. Although the ABT was performed before histological confirmation and before the initiation of surgery or chemotherapy, pancreatic cancer was already strongly suspected based on imaging at the time of testing. Therefore, disease-related changes in metabolic or general clinical status may have influenced the A/E ratio, and reverse causation cannot be excluded. Second, the health checkup group and patient groups were recruited from different institutions and during different time periods: health checkup participants were enrolled in 2017 at Izumi General Medical Center, whereas patients with benign pancreatic disease and pancreatic cancer were recruited between 2023 and 2025 at Kagoshima University Hospital. The health checkup participants were also substantially younger than the patient groups. Although we adjusted for age and other major covariates, residual confounding related to age distribution, calendar period, healthcare setting, referral patterns, and unmeasured background factors cannot be excluded. The associations remained statistically significant in an additional sensitivity analysis using health checkup participants aged ≥60 years as a more age-comparable reference group; however, this analysis did not eliminate potential residual confounding or selection bias related to differences in recruitment period, institution, and healthcare setting. Accordingly, the present findings should be interpreted as associations observed in a selected clinical study population rather than as estimates derived from a population-based case–control design. Health checkup participants did not undergo systematic pancreatic imaging; therefore, asymptomatic pancreatic cysts or other undiagnosed pancreatic abnormalities may have been present in this group, and some participants may have been misclassified as controls. Future multicenter studies using contemporaneous reference participants who are matched or otherwise balanced for age and recruited from comparable healthcare settings are needed to confirm these findings. Third, the benign pancreatic disease group included heterogeneous conditions that may have influenced the A/E ratio differently because of differences in disease pathophysiology, alcohol-related characteristics, inflammatory activity, and general clinical or metabolic status. Although additional post hoc analyses showed associations for both IPMN and non-IPMN benign pancreatic diseases, the non-IPMN group remained heterogeneous, and the small number of patients with each individual condition precluded reliable disease-specific multivariable analyses. Accordingly, the present findings cannot establish associations between the A/E ratio and individual benign pancreatic disorders. Fourth, the limited number of patients with pancreatic cancer raises concerns about statistical instability and potential model overfitting; although the direction of the association was consistent in the additional sensitivity analysis, the relatively wide confidence intervals indicate limited precision, and the effect estimates should be regarded as preliminary. Fifth, study-specific data on repeated-measure reproducibility, operator variability, and long-term instrument comparability were unavailable.

## 5. Conclusions

In this selected case–control study, higher ABT-derived A/E ratios were associated with benign pancreatic disease and pancreatic cancer after adjustment for major covariates. The categorized and spline analyses were exploratory and do not establish a clinically meaningful dose–response relationship, a validated threshold, or predictive utility. Prospective and disease-specific studies with external validation are required before the ABT-derived A/E ratio can be considered for pancreatic disease risk stratification.

## Figures and Tables

**Table 1:** Baseline characteristics.

	HC (n = 287)	Benign Pancreatic Diseases (n = 131)	Pancreatic Cancer (n = 65)	*p*-Value
Age, years (median, IQR)	49 (41–56)	71 (65–76)	73 (67–78)	<0.001
Male, n (%)	177 (61.7)	51 (38.9)	39 (60.0)	<0.001
BMI (median, IQR)	23.8 (21.5–26.5)	22.1 (19.9–24.6)	20.7 (19.0–23.5)	<0.001
Alcohol intake, g/day (median, IQR)	5.9 (0–24.2)	0.65 (0–16.3)	3.7 (0–43.9)	0.004
Alcohol category, n (%)				<0.001
0 g/day	74 (25.8)	62 (49.2)	27 (41.5)	
0–<46 g/day	181 (63.1)	47 (37.3)	22 (33.9)	
≥46 g/day	32 (11.1)	17 (13.5)	16 (24.6)	
Smoking (Brinkman index ≥ 600), n (%)	43 (15.0)	19 (14.5)	19 (29.2)	0.015
Diabetes mellitus, n (%)	15 (5.2)	25 (19.1)	27 (41.5)	<0.001
History of malignant disease, n (%)	10 (3.5)	14 (10.7)	14 (21.5)	<0.001
Family history of pancreatic cancer, n (%)	1 (0.3)	6 (4.6)	6 (9.2)	<0.001
A/E ratio (median, IQR)	13.6 (6.2–26.5)	21.7 (12.2–39.7)	24.2 (12.9–44.2)	<0.001
A/E ratio ≥ 23.3, n (%)	88 (30.7)	62 (47.3)	33 (50.8)	<0.001

**Table 2:** Multinomial logistic regression analysis of the association between ALDH2 inactive phenotype (A/E ratio ≥ 23.3) and pancreatic disease outcomes in the overall population and by sex.

	Overall		Male		Female	
	Benign Pancreatic Disease	Pancreatic Cancer	Benign Pancreatic Disease	Pancreatic Cancer	Benign Pancreatic Disease	Pancreatic Cancer
RRR (95% CI)	3.55 (1.85–6.83)	4.82 (2.13–10.88)	3.55 (1.33–9.43)	7.99 (2.42–26.41)	3.37 (1.36–8.36)	3.07 (0.92–10.19)

**Table 3:** Multinomial logistic regression analysis of the association between categorized A/E ratio and pancreatic disease outcomes.

	Benign Pancreatic Disease RRR (95% CI)	Pancreatic Cancer RRR (95% CI)
A/E ratio category		
<23.3	Reference	Reference
23.3–<36.9 (median)	1.68 (0.80–3.52)	2.04 (0.77–5.40)
≥36.9 (median)	8.77 (3.59–21.39)	12.79 (4.41–37.09)

RRR, relative risk ratio; CI, confidence interval; A/E ratio, acetaldehyde-to-ethanol ratio; BI, Brinkman Index. Multinomial logistic regression analysis was performed with the screening group as the reference category. The model was adjusted for age, sex, smoking status (Brinkman Index ≥ 600), alcohol consumption (g/day), and diabetes mellitus. A/E ratio categories were defined as <23.3, 23.3–<36.9 (median), and ≥36.9 (median). The median value (36.9) was calculated among participants with an A/E ratio ≥ 23.3.

## Data Availability

The study data are not deposited in a public repository because of participant privacy and ethical restrictions. De-identified data may be requested from the corresponding author and will be provided when the request is consistent with the applicable ethical and institutional requirements.

## Supplementary Materials

The following supporting information can be downloaded at https://www.mdpi.com/article/10.3390/jcm15197543/s1, Table S1: Sex-stratified baseline characteristics of the study population; Table S2: Distribution of specific diagnoses in the benign pancreatic disease group; Table S3: Additional analyses for IPMN and non-IPMN benign pancreatic diseases; Table S4: Sensitivity analysis using health checkup participants aged ≥60 years as the reference group.
